# Pharmacist participation in hospital ward teams and hospital readmission rates among people with dementia: a randomized controlled trial

**DOI:** 10.1007/s00228-017-2249-8

**Published:** 2017-04-08

**Authors:** Maria Gustafsson, Maria Sjölander, Bettina Pfister, Jeanette Jonsson, Jörn Schneede, Hugo Lövheim

**Affiliations:** 1 Department of Pharmacology and Clinical Neuroscience, Division of Clinical Pharmacology, Umeå University, SE-901 85 Umeå, Sweden; 2 Department of Community Medicine and Rehabilitation, Geriatric Medicine, Umeå University, Umeå, Sweden

**Keywords:** Medication reviews, Clinical pharmacists, Drug-related readmissions, Dementia, Old people

## Abstract

**Purpose:**

To assess whether comprehensive medication reviews conducted by clinical pharmacists as part of a healthcare team reduce drug-related hospital readmission rates among people with dementia or cognitive impairment.

**Methods:**

This randomized controlled trial was carried out between January 9, 2012, and December 2, 2014. Patients aged ≥65 years with dementia or cognitive impairment admitted to three wards at two hospitals located in Northern Sweden were included.

**Results:**

Of the 473 deemed eligible for participation, 230 were randomized to intervention and 230 to control group by block randomization. The primary outcome, risk of drug-related hospital readmissions, was assessed at 180 days of follow-up by intention-to-treat analysis.

During the 180 days of follow-up, 18.9% (40/212) of patients in the intervention group and 23.0% (50/217) of those in the control group were readmitted for drug-related reasons (HR = 0.80, 95% CI = 0.53–1.21, *p* = 0.28, univariable Cox regression). Heart failure was significantly more common in the intervention group. After adjustment for heart failure as a potential confounder and an interaction term, multiple Cox regression analysis indicated that pharmacist participation significantly reduced the risk of drug-related readmissions (HR = 0.49, 95% CI = 0.27–0.90, *p* = 0.02). A post-hoc analysis showed a significantly reduced risk of 30-day readmissions due to drug-related problems in the total sample (without adjustment for heart failure).

**Conclusion:**

Participation of clinical pharmacists in healthcare team conducting comprehensive medication reviews did not significantly reduce the risk of drug-related readmissions in patients with dementia or cognitive impairment; however, post-hoc and subgroup analyses indicated significant effects favoring the intervention. More research is needed. Trial registration: Clinical trials NCT01504672.

**Electronic supplementary material:**

The online version of this article (doi:10.1007/s00228-017-2249-8) contains supplementary material, which is available to authorized users.

## Introduction

Age-related changes such as renal impairment, comorbidities, and subsequent polypharmacy as well as drug-drug interactions pose challenges to appropriate pharmacotherapy in old people. Problems associated with drug treatment such as poor adherence, medication errors, and adverse drug events are common. Up to 30% of hospital admissions are related to drug-related problems (DRPs) among old people [1, 2] and an even higher proportion is seen among people with dementia [3]. Adverse drug reactions (ADRs), inappropriate drug use, drug-drug interactions, overprescription, or lack of required medication are contributing to drug-related hospital admissions [4]. Moreover, according to one meta-analysis, up to 24% of patients develop adverse drug reactions during their hospital stay [5].

Old people with dementia are particularly vulnerable to adverse drug reactions. A potential cause could be reduced acetylcholine levels in the brain compared to healthy individuals [6, 7]. Drug prescriptions are often not adapted to the special demands of patients with dementia [8]. Several studies indicate that use of potentially inappropriate drugs is common among this group of people [9, 10] despite the increased risk of adverse drug reactions and hospital admissions [11]. However, a high proportion of drug-related hospital admissions is preventable [12]. Often, multiple disciplines and specialties are involved in patient treatment with ill-defined distribution of responsibilities for the overall drug management. Participation of a clinical pharmacist in the multidisciplinary hospital ward team could ensure a more coherent pharmacotherapeutic approach across the traditional borders of medical specialities and thereby reduce the risk of DRPs [13].

Recent systematic reviews suggest that interventions by clinical pharmacists can improve patient outcomes in both inpatient and outpatient care facilities [14–17]. However, the results are inconsistent whether pharmacist interventions can reduce hospital readmissions and mortality [18]. In many countries such as the USA, clinical pharmacists have been a natural part of the multiprofessional health care teams for many years [19].

To the best of our knowledge, the effectiveness of clinical pharmacist participation in a ward team on the risk of hospital readmissions in individuals with dementia or cognitive impairment has yet not been studied. The aim of the present study is to assess whether comprehensive medication reviews conducted by clinical pharmacists as members of a ward team could reduce the rate of drug-related hospital readmissions among old people with dementia or cognitive impairment.

## Methods

### Study design, setting, and participants

A randomized controlled study design was used to compare hospitalized patients obtaining usual care with those receiving additional standardized medication reviews performed by an experienced clinical pharmacist. Patients admitted to acute internal medicine wards at the Skellefteå County Hospital (*n* = 108) and Umeå University Hospital (*n* = 290) and to the orthopedic ward at Umeå University Hospital (*n* = 62) were included. These were selected on the basis of being the wards where the clinical pharmacists already worked at study start. Both hospitals are located in Northern Sweden.

Eligible patients were aged 65 years or older and had dementia or cognitive impairment. Medical records were carefully reviewed before inclusion to avoid the risk of including people without dementia or cognitive impairment. Dementia diagnoses were collected from the medical records. Patients were considered to have cognitive impairment if sufficient information in the medical record related to memory, orientation, or executive function was noted before index hospitalization. In addition, patients in whom dementia was suspected and medical investigation had been commenced or would be initialized were included. In ambiguous or uncertain cases, patients were excluded.

### Ethical approval

In Sweden, the Ethical Review Law permits research involving persons with cognitive impairment under certain conditions, even though they cannot give a full informed consent. The procedure still should be as “informed consent-like” as possible taking into account the cognitive level of the persons. The permission for the present study was sought and approved for research without consent in accordance with the Swedish Ethical Review Law (Regional Ethical Review Board in Umeå, Sweden, registration number 2011-148-31M). Research person and their next of kin were given written and orally presented information about the research, individually adjusted to their cognitive level, and persons who did not wish to participate were able to decline or withdraw from the study.

### Randomization and masking

The patients were randomly assigned to one of two groups: intervention group or control group. The randomization sequence was prepared before study start using a throwing dice—method by an independent person who was not engaged in the trial in any other way. The sequence was performed in blocks of 6–36 (each block contained between 3 and 18 intervention allocations and the same number of control allocations). Randomization was stratified at ward level. To accomplish this, each ward used their own randomization blocks, consecutively starting a new block after completion of the preceding, meaning that there were an equal number of control and intervention participants in each ward.

When a patient formally entered the trial, an employee of the Department of Pharmacology and Clinical Neuroscience who was not involved in the interventions provided the treatment allocation according to the randomization scheme. The patients and pharmacists were not blinded to treatment assignment.

### Intervention

Three clinical pharmacists with post-graduate degrees in clinical pharmacy and long experience in performing medication reviews in primary care and hospital wards conducted the interventions. The pharmacists were already part of the different ward teams at the time when the study started. The additional service provided by the clinical pharmacists consisted of medication reconciliation, medication review, and participation in ward rounds. The three clinical pharmacists met continuously throughout the study period and discussed interventions to harmonize the advices given during ward rounds.

By conducting medication reconciliation, the pharmacists ensured that the medication administration records used at the wards were updated, accurate, and complete. Various information sources were used, including drug lists from primary care centers, the patients’ hospital medical records, and in two cases, interviews with patients and/or relatives.

Based on an updated drug list, a comprehensive medication review was performed by the clinical pharmacist comprising aspects associated with the patients’ drug therapy, including the medication list, list of laboratory results, medical record notes from primary care and index admission, and also notes from earlier contacts with healthcare providers, to compile an extensive medication history. In addition, general data regarding age, gender, and patient history were collected. All data were recorded on a patient-specific documentation sheet. The clinical pharmacists identified relevant DRPs with respect to impairment of body function (renal function, liver function, contraindications, allergies, swallowing problems), certain drug use (toxic drugs, drugs prone to produce side effect, potentially inappropriate drugs), interactions (drug-drug, and drug-food), symptoms (adverse drug reactions), and general judgment of the patient’s drug use (proper drug selection, dosage, duration of treatment, polypharmacy, indication for therapy, untreated indication, adherence, over-the-counter drugs, and effectiveness). Clinical response to drug treatment was monitored throughout the hospital stay.

The clinical pharmacist participated in ward rounds, and clinically relevant DRPs were discussed with the healthcare team (physicians, nurses, enrolled nurses). Advice was given about drug selection, dosages, and possible monitoring needs. The attending physicians made the final decision concerning proposed changes to therapy. The acceptance or rejection of the pharmacist’s recommendation for changes in drug therapy was documented. All DRPs were recorded on a standardized form and classified according to Cipolle et al. [20] into seven categories: unnecessary drug therapy, needs additional drug therapy, ineffective drug, dosage too low, dosage too high, adverse drug reactions, and non-adherence. The follow-up time was 180 days after discharge from index admission.

### Outcomes

To assess the primary outcome, risk of drug-related readmissions, data were collected from electronic medical records during the first 180 days after discharge from index admission. An independent, blinded external expert group consisting of one specialist in geriatrics, one specialist in internal medicine, and one clinical pharmacist working in another county assessed the outcomes. For each participant, the expert group received the drug list, laboratory list, doctors’ notes, and epicrisis from the first admission and from any readmission(s). Data were copied from the medical records and carefully reviewed twice to make sure anything that could reveal group assignment was deleted. Before handed to the experts, data were also anonymized.

The expert group decided whether the readmissions were to be considered drug-related or not. They were instructed to focus on all sorts of problems concerning drug treatment, i.e., problems actually caused by a prescribed drug, but also problems with not having a drug prescribed or adherence problems. Discordant judgments were referred for consensus discussions in the whole expert group to reach a decision. In those cases where obvious suspected drug-related problems were found by the clinical pharmacists, the information were given back to the expert group for a second valuation (still blinded). The likelihood that readmissions were drug-related was graded into categories *certain*, *probable*, *possible*, or *unlikely/un-assessable*, in accordance with the World Health Organization (WHO) criteria for causality assessment of ADR [21]. Later, in statistical analysis, readmissions classified as *certain*, *probable*, and *possible* were grouped as “drug-related”, the remaining as “non-drug-related”. A secondary outcome parameter was “all-cause” readmission. At the time the study was planned in 2011, a follow-up period of 180 days for hospital readmissions was considered adequate. However, in late 2012, the hospital readmission reduction program was launched in the USA and England restricting payments for early readmissions within 30 days of discharge from a previous (index) admission [22]. Consequently, we also evaluated short-term effects of the intervention (readmissions within 30 days) in a post-hoc analysis. Secondary outcomes included cost analysis, time to institutionalization, and adherence to quality indicators (not yet analyzed).

### Statistical analyses

We calculated that a sample size of 460 patients would provide 80% power to detect a 20% reduction in readmissions attributed to the participation of a clinical pharmacist. An intention-to-treat analysis was performed, including all participants except those who died during the hospital stay before discharge (no follow-up time). For analysis of the primary outcome parameter, a Cox regression model was used.

There was a significant difference in the prevalence of heart failure between the intervention and control group. Heart failure had a significant impact on the risk of readmission, and furthermore, the intervention did not have any effect among those with heart failure. These confounding and interaction effects were accounted for by including a heart failure and an interaction term between “intervention” and “heart failure” in the final model.

First-time drug-related readmissions after index discharge were summarized in Kaplan-Meier curves separately for the intervention and the control group applying a log rank test. Dichotomous variables were analyzed using the Pearson chi-square test and continuous variables using the independent sample *t* test. Differences between the groups for the number of readmissions were analyzed applying the Mann-Whitney *U* test. IBM SPSS Statistics package v.22.0 was used for statistical analyses. We regarded *p* values of 0.05 or less to be statistically significant.

## Results

**Fig. 1:**
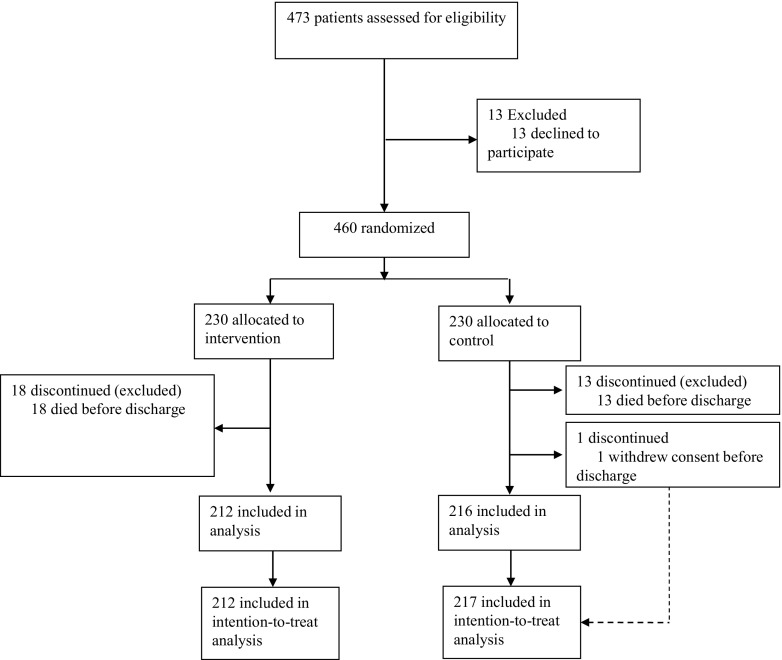
Patient flow chart throughout the study

Between January 9, 2012, and December 2, 2014, 473 patients aged 65 years or older were invited to participate in the trial. Thirteen subjects declined participation. The remaining 460 patients were randomized (230 to the intervention group and 230 to the control group). One individual in the control group used the right to withdraw from the trial before discharge. In addition, 31 patients (18 in intervention and 13 in control) died before discharge. These 31 individuals were excluded from the analysis, leaving a final sample of 429 patients. Figure 1 illustrates the patient flow throughout the trial. No significant differences between the intervention and control group were found for the majority of baseline characteristics. However, significantly more patients in the intervention group had a history of heart failure compared to the control group (34 vs 25%, *p* = 0.04) (Table 1).

**Table 1:** Baseline characteristics of participants randomized to control or intervention groups

	Control (*n* = 217)	Intervention (*n =* 212)	*p* value
Women	138 (64%)	133 (63%)	0.854
Age, mean (SD), years	83.1 (6.6)	83.1 (6.6)	0.996
Laboratory values			
Sodium level, mean (SD), mmol/L	139.1 (4.1)	138.9 (5.1)	0.568
Potassium level, mean (SD), mmol/L	4.1 (0.5)	4.1 (0.5)	0.774
Hb, mean (SD), g/L	123.6 (19.1)	124.7 (17.6)	0.515
Creatinine clearance, mean (SD), mL/min^a^	56.8 (23.1)	53.6 (21.9)	0.145
Duration of index admission, mean (SD), days	9.1 (7.9)	8.3 (7.2)	0.302
Drugs, mean (SD), number	8.3 (3.6)	8.4 (3.6)	0.622
Type of living, no. (%)			0.369
Living at home	158 (73)	146 (69)	
Nursing home	59 (27)	66 (31)	
Dementia subtype, no. (%)			
Alzheimers disease	68 (31)	64 (30)	0.797
Vascular dementia	30 (14)	42 (20)	0.097
Other or unspecified dementia	119 (55)	106 (50)	0.316
MMSE, mean (SD)^b^	20.1 (4.3)	19.6 (4.8)	0.537
Medical history, no. (%)			
Heart failure	54 (25)	72 (34)	0.039
Hypertension	105 (48)	116 (55)	0.190
Cardiac arrhythmia	58 (27)	62 (29)	0.561
Diabetes mellitus	47 (22)	61 (29)	0.090
Chronic obstructive pulmonary disease	18 (8)	16 (8)	0.774
Malignant disease, past or present	20 (9)	27 (13)	0.243
Myocardial infarction, past	25 (12)	36 (17)	0.105
Stroke, past	46 (21)	50 (24)	0.533

Figures are numbers of participants (percentage) unless stated otherwise

*MMSE* mini mental state examination, *Hb* hemoglobin

^a^Creatinine clearance was estimated from plasma creatinine values using the Cockcroft-Gault equation

^b^Data missing for 154 patients in the control group and 119 patients in the intervention group

The clinical pharmacists identified at least one DRP in 66% (140/212) of individuals in the intervention group, summing up for a total of 310 DRPs. The doctors followed the advice of the clinical pharmacists in 82% of the identified DRPs (74% of proposed actions were already effectuated during the hospital stay while 8% were issued as written recommendations in the discharge notes addressed to the general practitioners). Actions taken to the suggested DRPs were discontinuation of drug therapy (*n* = 78), followed by reduction in dosage (*n* = 45) and correction of transition errors (*n* = 22). Initiation of drug therapy (*n* = 21), change of drug (*n* = 19), monitoring of laboratory values (*n* = 13), increase in dosage (*n* = 8), and change of drug formulation (*n* = 4) were other actions taken for the clinical pharmacists’ suggestions. Further, 20 actions taken were categorized as “other”, 24 suggestions were written in discharge notes, and 56 of the suggestions were rejected.

The DRPs were classified as follows: ADR (*n* = 103), ineffective drug/inappropriate drug (*n* = 54), unnecessary drug therapy (*n* = 54), dosage too high (*n* = 44), needs additional drug therapy (*n* = 37), dosage too low (*n* = 14), and non-adherence (*n* = 4). The time spent on performing a medication review was on average 32 min per patient (range 10–90 min). Approximately 20 min per patient was spent in ward rounds, and it took 10 min for the clinical pharmacists to walk to the ward and back, in total, 62 min.

**Fig. 2:**
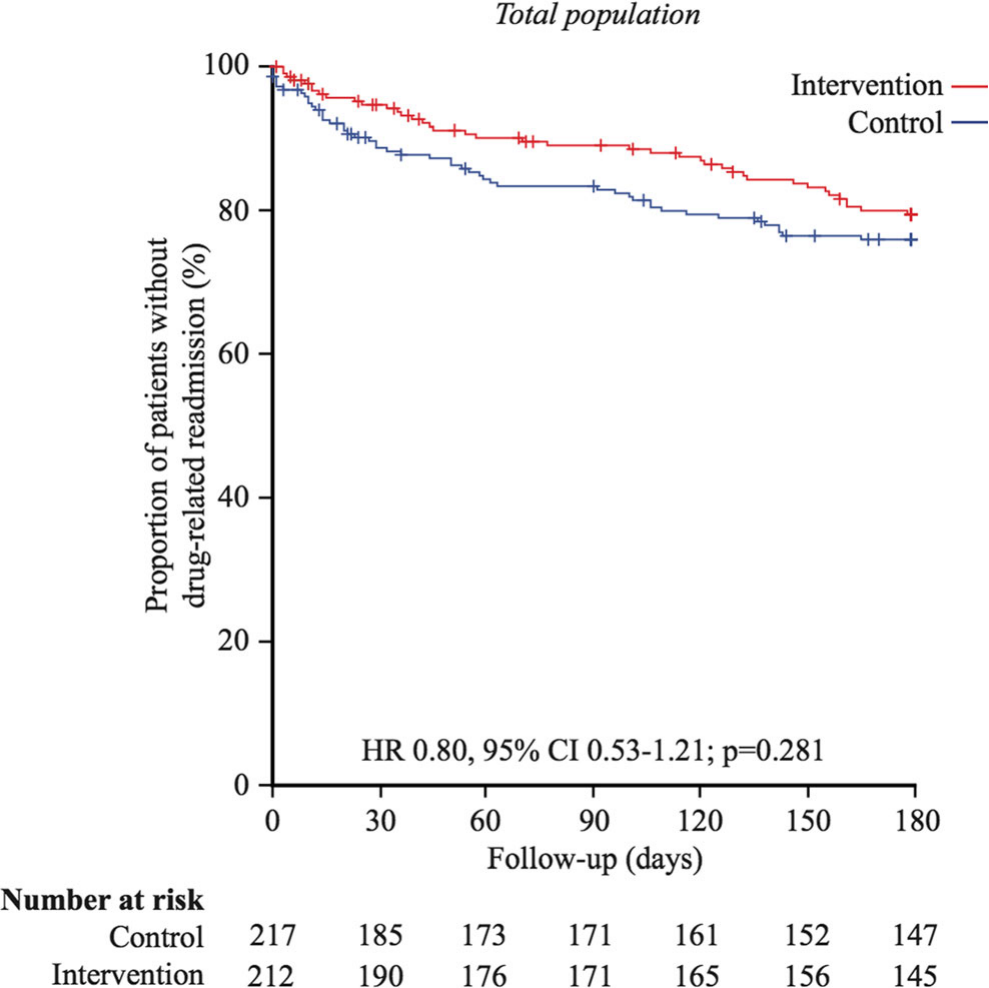
Kaplan-Meier plots for drug-related readmissions within 180 days in the total sample. HR and CI according to univariable Cox regression analysis and *p* value from log rank test

The frequencies of readmissions and deaths during the 180 days of follow-up after discharge are summarized in Table 2. During this period, 18.9% (40/212) of patients in the intervention group and 23.0% (50/217) of patients in the control group were readmitted for drug-related reasons (HR 0.80, 95% CI 0.53–1.21, *p* = 0.28, univariable Cox regression). A Kaplan-Meier survival analysis showed no significant difference in time-to-drug-related readmission within 180 days between the intervention and control groups (160.0 (standard deviation 3.3) days vs 150.1 (4.0) days, Mantel-Cox log rank test, *p* = 0.28) (Fig. 2). Heart failure was significantly more common in the intervention group (*p* = 0.04) and was associated with an increased risk of drug-related readmissions (HR 2.48, 95% CI 1.64–3.76, *p* < 0.001). Pharmacist intervention had no impact on drug-related readmissions among patients with heart failure (HR 1.16, 95% CI 0.63–2.15, *p* = 0.64). Inclusion of heart failure as a confounder and an interaction term between heart failure and the intervention in a multiple Cox regression model revealed that after adjustment for heart failure, the intervention significantly reduced the risk of drug-related readmissions (HR 0.49, 95% CI 0.27–0.90, *p* = 0.02).

**Table 2:** Outcomes at 30- and 180-day follow-up, total sample

	Control (*n* = 217)	Intervention (*n* = 212)	*p* value
Drug-related readmissions			
Drug-related readmissions, no.	68	58	0.32
Certain (no. of individual patients^a^)	3 (3)	3 (3)	
Probable (no. of individual patients^a^)	25 (22)	24 (16)	
Possible (no. of individual patients^a^)	40 (25)	31 (23)	
Patients readmitted because of DRP, no. (%)	50 (23)	40 (19)	0.29
Patients readmitted because of DRP within 30 days, no. (%)	24 (11)	11 (5)	0.03
Readmissions all causes			
Patients readmitted, no. (%)	88 (41)	81 (38)	0.62
Readmissions, no.	141	138	0.62
Patients readmitted within 30 days, no. (%)	40 (18)	31 (15)	0.29
Mortality			
Patients deceased	34 (16%)	44 (21%)	0.17

*DRP* drug-related problems

^a^The same person might have more than one type of drug-related readmission

**Fig. 3:**
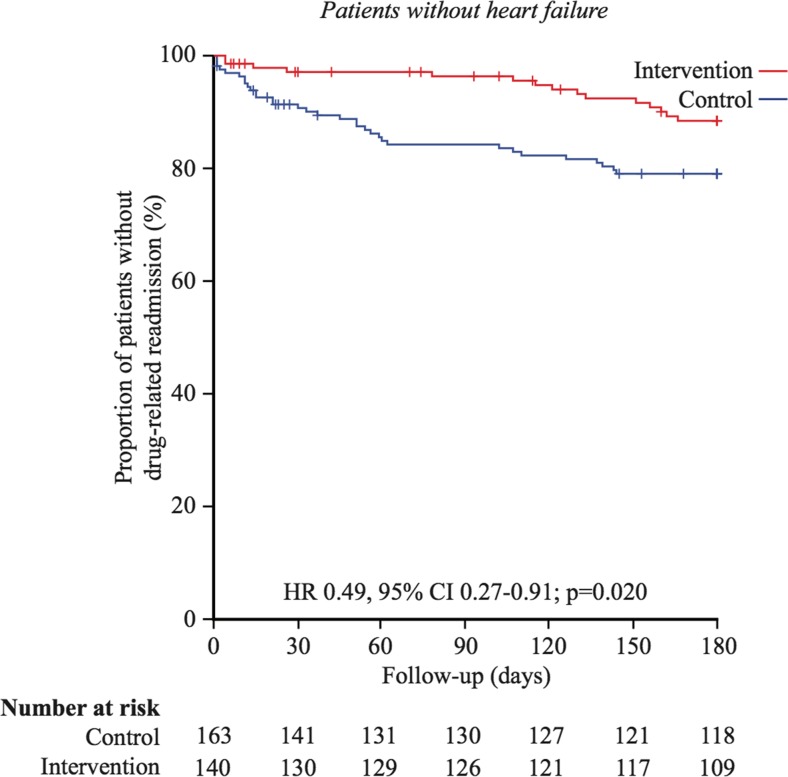
Kaplan-Meier plots for drug-related readmissions within 180 days in the subgroup of people without heart failure. HR and CI according to univariable Cox regression analysis and *p* value from log rank test

Subgroup analyses among patients without heart failure were performed. In this subgroup (140 intervention and 163 control), the 180-day drug-related readmission rate was significantly lower in the intervention group than that in the control group; 11% (15/140) and 20% (33/163), in the intervention and the control group, respectively (*p* = 0.02) (Table 3). A Kaplan-Meier survival analysis showed that the time-to-drug-related readmission within 180 days was significantly longer in the intervention group than that in the control group (171.2 (2.7) days vs 153.1 (4.5) days, Mantel-Cox log rank test, *p* = 0.02) (Fig. 3).

**Table 3:** Outcomes at 30- and 180-day follow-up, total sample without heart failure

	Control (*n* = 163)	Intervention (*n* = 140)	*p* value
Drug-related readmissions			
Drug-related readmissions, no.	46	23	0.03
Certain (no. of individual patients^a^)	1 (1)	0 (0)	
Probable (no. of individual patients^a^)	13 (12)	7 (6)	
Possible (no. of individual patients^a^)	32 (20)	16 (10)	
Patients readmitted because of DRP, no. (%)	33 (20)	15 (11)	0.02
Patients readmitted because of DRP within 30 days, no. (%)	15 (9)	4 (3)	0.02
Readmissions all causes			
Patients readmitted, no. (%)	60 (37)	41 (29)	0.17
Readmissions, no.	92	66	0.17
Patients readmitted within 30 days, no. (%)	27 (17)	14 (10)	0.10
Mortality			
Patients deceased, no. (%)	21 (13)	20 (14)	0.72

*DRP* drug-related problems

^a^The same person might have more than one type of drug-related readmission

**Fig. 4:**
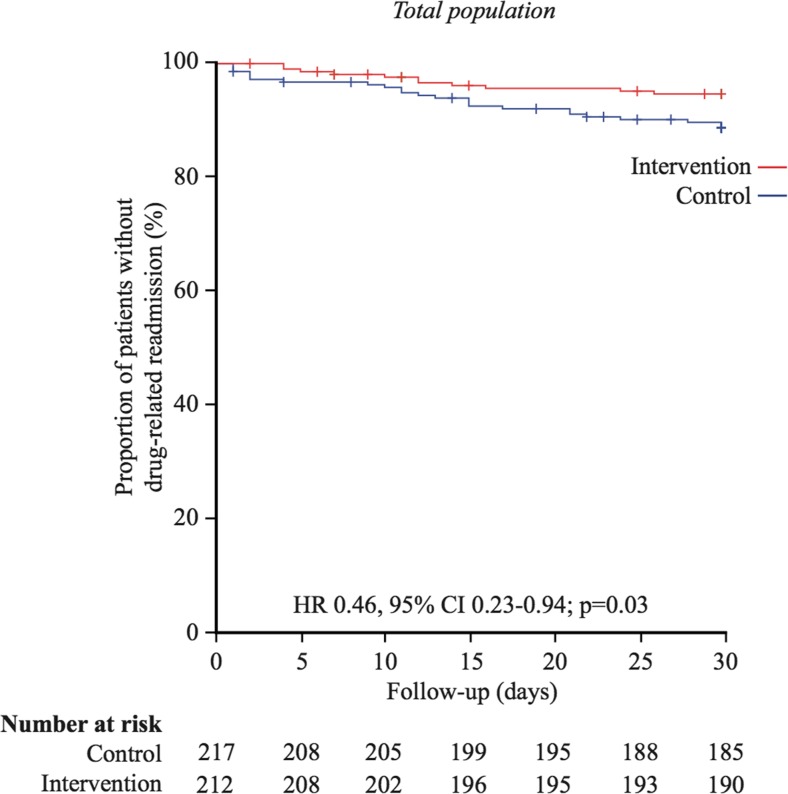
Kaplan-Meier plots for drug-related readmissions within 30 days in the total sample. HR and CI according to univariable Cox regression analysis and *p* value from log rank test

**Fig. 5:**
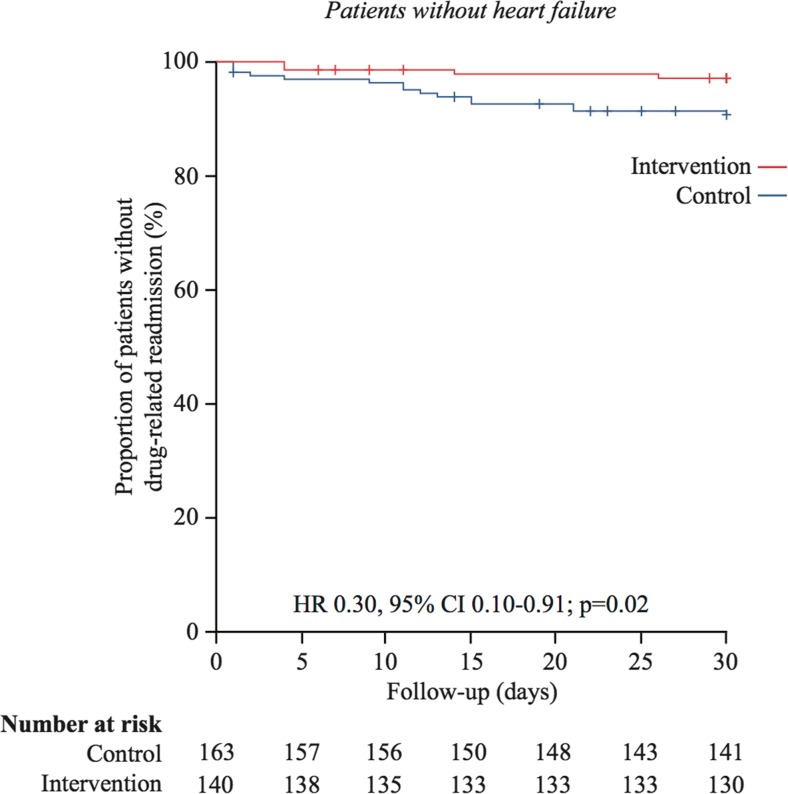
Kaplan-Meier plots for drug-related readmissions within 30 days in the subgroup of people without heart failure. HR and CI according to univariable Cox regression analysis and *p* value from log rank test

Additional analyses of the risk of early readmissions (≤30 days) were performed. We observed a significant difference in the frequency of DRP readmissions within 30 days between the intervention group (5% (11/212)) and the control groups (11% (24/217)), *p* = 0.03) in the total study population (including patients with heart failure) (Table 2). Moreover, Kaplan-Meier curve analyses revealed significant differences in time to drug-related readmission during the first 30 days after discharge between the intervention and the control group in the total study population (29.1 (0.30) days vs 28.1 (0.43) days, Mantel-Cox log rank test, *p* = 0.03) (Fig. 4) and among patients without heart failure (29.5 (0.29) days vs 28.3 (0.49) days, Mantel-Cox log rank test, *p* = 0.02) (Fig. 5). Further, sensitivity analyses were done, using certain and probable (but not possibly) drug-related readmissions. After adjustment for heart failure as a potential confounder and an interaction term, a multiple Cox regression analysis showed no difference between the groups (HR = 0.46, 95% CI = 0.18–1.18, *p* = 0.10). In Appendix 1, the other main analyses for certain and probable (but not possibly) drug-related readmissions are presented.

## Discussion

We found that the intervention did not significantly reduce the risk of drug-related readmissions at 180 days of follow-up. However, after adjustment for heart failure, the intervention significantly reduced the risk and further, in a post-hoc analysis of early readmissions, a significantly reduced risk of 30-day readmissions due to DRPs was observed in the total sample (without adjustment for heart failure). There were also a lower number of all-cause early readmissions in the intervention group, but the difference between the groups did not reach statistical significance. As sensitivity analyses, main analyses were repeated using only certain and probable (but not possibly) drug-related readmissions. The results, as presented in Appendix 1, were in general similar to the results calculated for certain, probable, and possibly drug-related readmissions concerning estimated hazard ratios, but did not reach significance due to the lower number of readmissions and thereby lack of power.

A recent systematic review concluded that medication reviews performed by clinical pharmacists at hospitals may improve patient outcome [16], although another review investigating interventions performed by different health care professionals did not show effects on readmission and mortality [18]. However, in this review, also type of intervention differed between the included studies [18]. One randomized controlled study not included in the review mentioned above demonstrated a significant reduction in all cause readmissions and increased time to readmission among individuals aged 65 years and older [23]. Moreover, Gillespie et al. revealed a significant reduction in drug-related readmissions among participants aged 80 years and older [4]. In these two studies, clinical pharmacists conducted comprehensive medication reviews, in the same way as in the present study.

In our study, no effect of the pharmacist intervention was observed in patients with concomitant heart failure. This contrasts findings of a systematic review indicating that participation of a pharmacist in a multidisciplinary heart failure team may reduce the rate of all-cause and heart failure readmissions by almost one-third [24]. However, patients in our study were cognitively impaired. Adherence to medication is crucial for treatment of patients with heart failure, [25] and some of the patients in our study were readmitted particularly because of adherence problems. Most of the people lived at home, and many patients were unable to understand the need for liquid restrictions or diuretic dosing self-adjustments. Moreover, some patients had not been taking their heart failure medication at all.

Heart failure is a severe clinical condition with high risk of exacerbations. Frequent readmissions for medication adjustments may be a requisite for proper follow-up and need not necessarily indicate poor quality of care [22]. Our finding that pharmacist participation did not influence the readmission rate among cognitively impaired patients with heart failure should be seen against the background of the severity of the condition. Nevertheless, according to Koshman et al. [24], participation of clinical pharmacists can still be beneficial to this group; however, the mode of intervention possibly needs to be revised. Face-to-face meetings between the pharmacist and the patient could be important for adherence [4]. In a comprehensive approach, involvement of relatives in the patients’ drug therapy may be necessary.

Still, 30-day readmission rates are increasingly being used as an indicator of quality of care [26, 27]. Our finding that participation of a pharmacist in a ward team may significantly reduce the risk of early readmissions due to DRPs is important not only in this context. Let alone the risk of hospital penalization in 30-day readmissions [22], and the costs associated with avoidable readmissions, hospitalized individuals also are at an increased risk of worsening of the general health status and may develop confusion or complications from immobility [28].

In the present study, the physicians followed the advice of the clinical pharmacists to a high degree, 82%. This high rate should be seen in the context that the three pharmacists performing the intervention were already acknowledged by the healthcare teams and had been working at the wards before the trial. Close collaboration between the pharmacist and the prescriber is crucial for a successful intervention. [13, 29] A recent study from Denmark investigated the impact of medication reviews performed by a pharmacist and a clinical pharmacologist at an orthopedic ward without being part of the healthcare team. Here, the acceptance rate was only about 18%, and the intervention had no effect on clinical outcome [30].

In the present study, pharmacists had full access to medical and laboratory records and the intervention comprised medication reconciliation, comprehensive medication reviews, and communication of findings at ward rounds. The comprehensiveness of the medication review and feedback during ward rounds may have contributed to the significant effect (post-hoc and subgroup analyses) on readmission rates.

### Limitations

The study has some limitations that should be considered. The 30-days readmission analysis was not pre-specified in the study protocol. However, because of the increased use of 30-day readmission as an indicator of quality of care, the outcome was added as a post-hoc analysis after the study was started.

The clinical pharmacists engaged in this study were highly experienced and had been working for up to 8 years at the respective wards in the present study. This, together with the fact that patients from the same wards were randomized to both the intervention and the control group, may have caused a risk of contamination bias. During the study period, the clinical pharmacists worked not only with study participants but also with other patients in the wards. The prescribing physician and the clinical pharmacist discussed DRPs of both those in the intervention group and those not included in the study, and it is not unreasonable to assume that after the reviews, the physicians might have transferred knowledge to the control patients. Therefore, it is possible that the intervention would have had a higher impact if the clinical pharmacists had not been working in the wards before the intervention, and if the intervention and control patients were located in different wards. However, the design of the study reflects a real-life setting, and results indicate that the intervention had an effect even though the pharmacists originally worked in the selected wards.

We did not evaluate if the DRPs identified by the clinical pharmacists were clinically relevant and significant. However, based on the high acceptance rate (82%), it is reasonable to assume that most of the DRPs were judged to be clinically relevant by the physician in charge.

Another caveat is that the primary outcome parameter, drug-related readmissions, is not an objective measure. To compensate for this, and in order to capture all aspects of DRPs, individuals with different professional backgrounds were recruited for the independent consensus group, and all members were unaware of treatment assignments. The reasons for hospitalization are in many cases multifactorial, and DRPs are often only one of several factors leading to admission. Most of the drug-related readmissions were classified as *possibly* contributing to readmission, likely because of this.

In this study, the effects of pharmacist-led comprehensive medication reviews were investigated. Whether or not the results would have been different if the medication reviews had been performed by someone not being a clinical pharmacist was not the scope of this specific study.

### Conclusion

Comprehensive approaches and interdisciplinary collaboration are needed to avoid unnecessary hospitalizations and early readmissions among people with dementia. Participation of clinical pharmacists in healthcare team conducting comprehensive medication reviews did not significantly reduce the risk of drug-related readmissions in patients with dementia or cognitive impairment; however, post-hoc and subgroup analyses indicated significant effects favoring the intervention. These findings need confirmation in future studies.

## Electronic supplementary material

ESM 1 (DOCX 437 kb)
