# Supplementary material for: Pharmacist participation in hospital ward teams and hospital readmission rates among people with dementia: a randomized controlled trial
Source: Eur J Clin Pharmacol. 2017 Apr 8;73(7):827–35. doi: 10.1007/s00228-017-2249-8 (PMC5486919; doi:10.1007/s00228-017-2249-8)
Supplement: Supplementary file 1 — (DOCX 437 kb) [file 228_2017_2249_MOESM1_ESM.docx]

Pharmacist participation in hospital ward teams and hospital readmission rates among people with dementia: a randomized controlled trial

## Appendix 1

European Journal of Clinical Pharmacology

Maria Gustafsson, PhD^1,2^, Maria Sjölander, PhD^1^, Bettina Pfister, MPharm^1^, Jeanette Jonsson, MSc Pharm^1^ , Jörn Schneede, PhD^1^ , Hugo Lövheim, PhD^2^

^1^ Department of Pharmacology and Clinical Neuroscience, Division of Clinical Pharmacology, Umeå University, Umeå, Sweden

^2^ Department of Community Medicine and Rehabilitation, Geriatric Medicine, Umeå University, Umeå, Sweden

**Corresponding Author:**

Maria Gustafsson, Department of Pharmacology and Clinical Neuroscience, Umeå University, Umeå, Sweden, SE-901 87 Umeå, Sweden. Phone: +46 90 785 35 62;

Fax: +46 90 12 04 30

E-mail: [maria.gustafsson@umu.se](mailto:maria.gustafsson@umu.se)
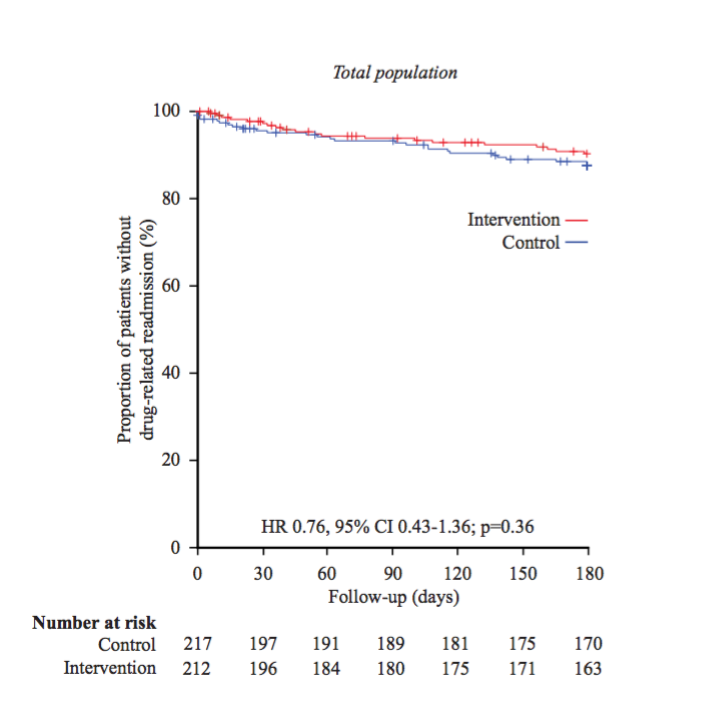


**Figure 1** Kaplan-Meier plots for drug-related readmissions (certain and probable) within 180 days in the total sample. HR and CI according to univariable Cox regression analysis and p-value from log rank test.

HR and CI according to univariable Cox regression analysis and p-value from log rank test.


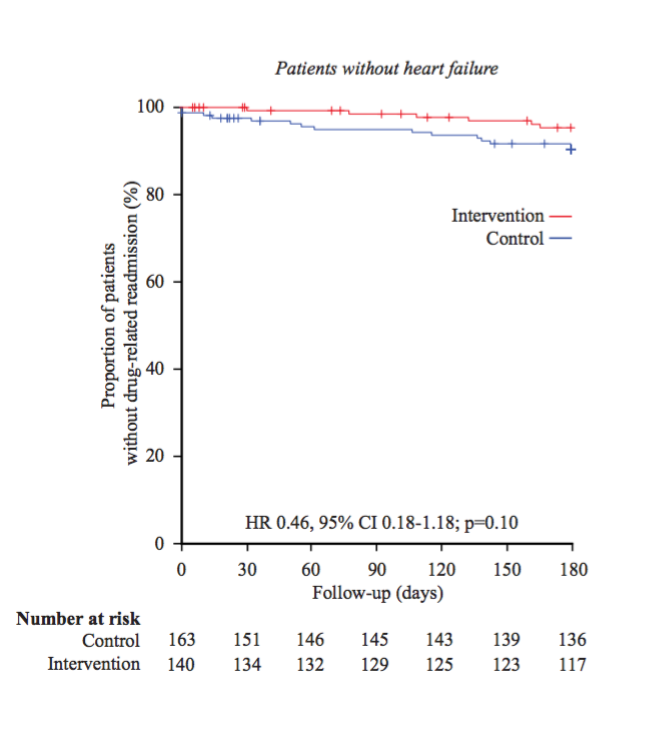


**Figure 2** Kaplan-Meier plots for drug-related readmissions (certain and probable) within 180 days in the subgroup of people without heart failure. HR and CI according to univariable Cox regression analysis and p-value from log rank test.

HR and CI according to univariable Cox regression analysis and p-value from log rank test.


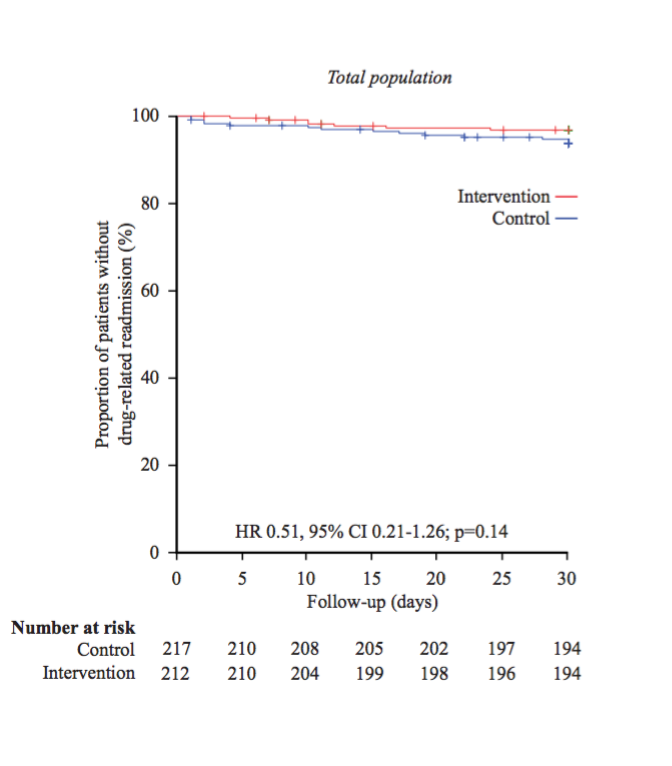


**Figure 3** Kaplan-Meier plots for drug-related readmissions (certain and probable) within 30 days in the total sample. HR and CI according to univariable Cox regression analysis and p-value from log rank test.

HR and CI according to univariable Cox regression analysis and p-value from log rank test.


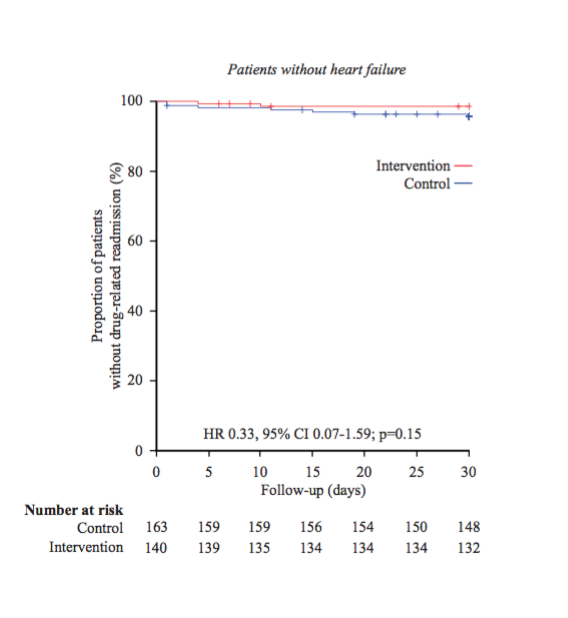


**Figure 4** Kaplan-Meier plots for drug-related readmissions (certain and probable) within 30 days in the subgroup of people without heart failure. HR and CI according to univariable Cox regression analysis and p-value from log rank test.

HR and CI according to univariable Cox regression analysis and p-value from log rank test.
